# Transoral penetrating craniocerebral injury: a case report and literature review

**DOI:** 10.3389/fsurg.2024.1455178

**Published:** 2024-11-08

**Authors:** Hang Xue, Wei Li, Weitao Zhang, Lei Sun, Yubo Li, Hongfa Yang

**Affiliations:** ^1^ Department of Neurotraumatic Surgery, The First Hospital of Jilin University, Changchun, Jilin, China; ^2^ Department of Neurosurgery, Yantaishan Hospital Affiliated to Binzhou Medical University, Yantai, Shandong, China; ^3^ The Key Laboratory of Pathobiology, Ministry of Education, The College of Basic Medical Sciences, Jilin University, Changchun, Jilin, China; ^4^ Department of Neurosurgery, Jiutai District People's Hospital, Changchun, Jilin, China

**Keywords:** arrow, suicide, transoral, penetrating craniocerebral injury, surgery

## Abstract

A penetrating brain injury is a rare type of neurosurgical trauma associated with extremely high mortality and disability rates. Penetrating skull base injuries caused by arrows seldom occur because injuries caused by such weapons are more likely to be accidental. However, the number of self-inflicted injuries is increasing, and these injuries have varying patterns and high mortality rates. We report a case of a transoral penetrating craniocerebral injury caused by an arrow in a suicidal patient. Preoperative imaging is crucial for detecting and planning the surgical approach. Surgery is an effective treatment for this type of injury. Additionally, we reviewed previous case reports on this type of injury to provide recommendations for its clinical detection and treatment.

## Introduction

Transoral penetrating craniocerebral injuries are a rare complication of neurosurgery, and their primary treatment is craniotomy due to the presence of a foreign body. Craniocerebral CT is the main imaging method for this condition, and cranial reconstruction is an important basis for formulating relevant surgical protocols. Because the incidence of postoperative intracranial infection is extremely high, antibiotics are necessary to prevent and treat infection.

## Case description

**Figure 1:**
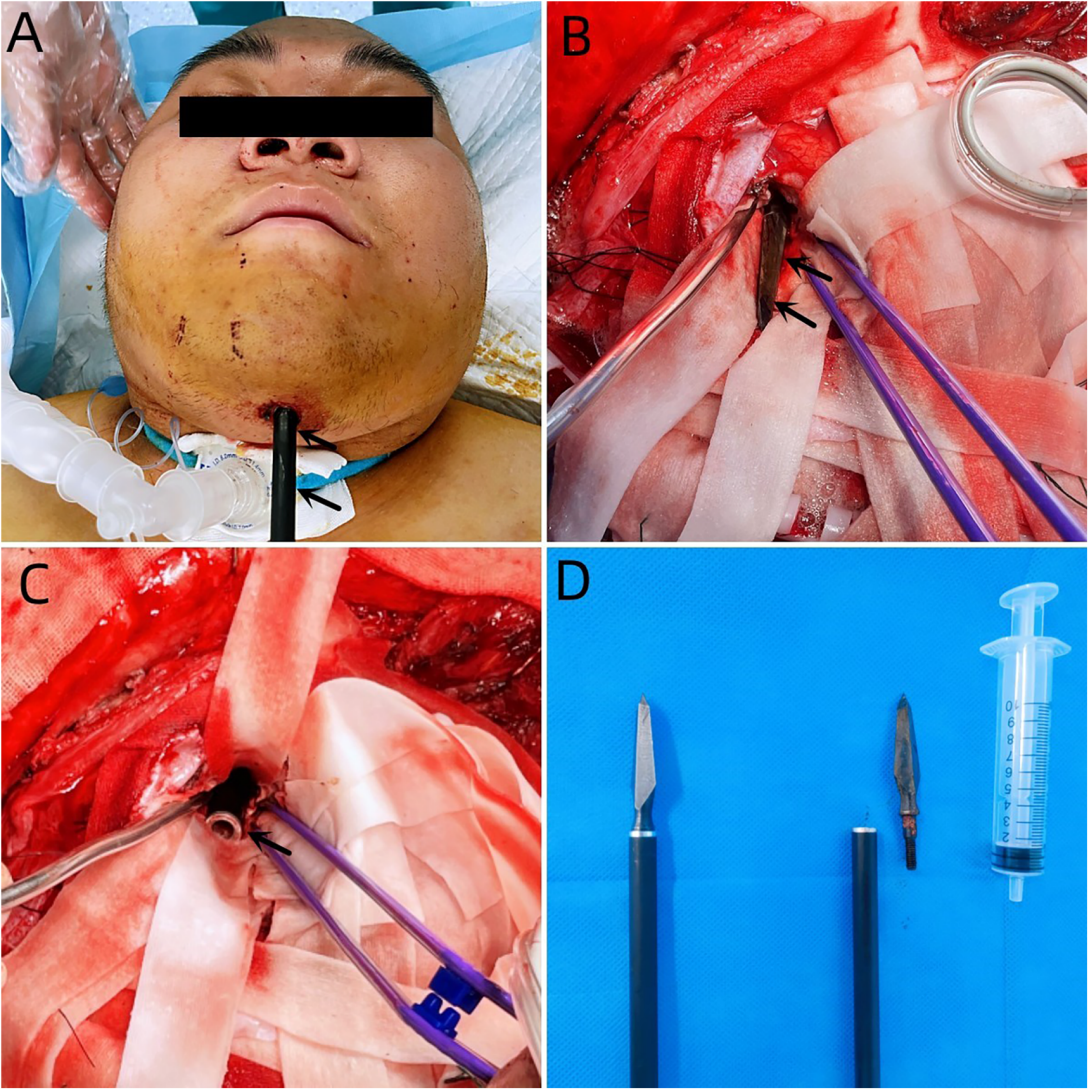
**(A)** Penetration of the skin of the mandibular region with an arrow, with its tail outside the body. **(B)** The black arrow indicates the head of the arrow. **(C)** The black arrow indicates the tail of the arrow. **(D)** The right image shows the intraoperative removal of the arrow, and the left image shows the arrow.

**Figure 2:**
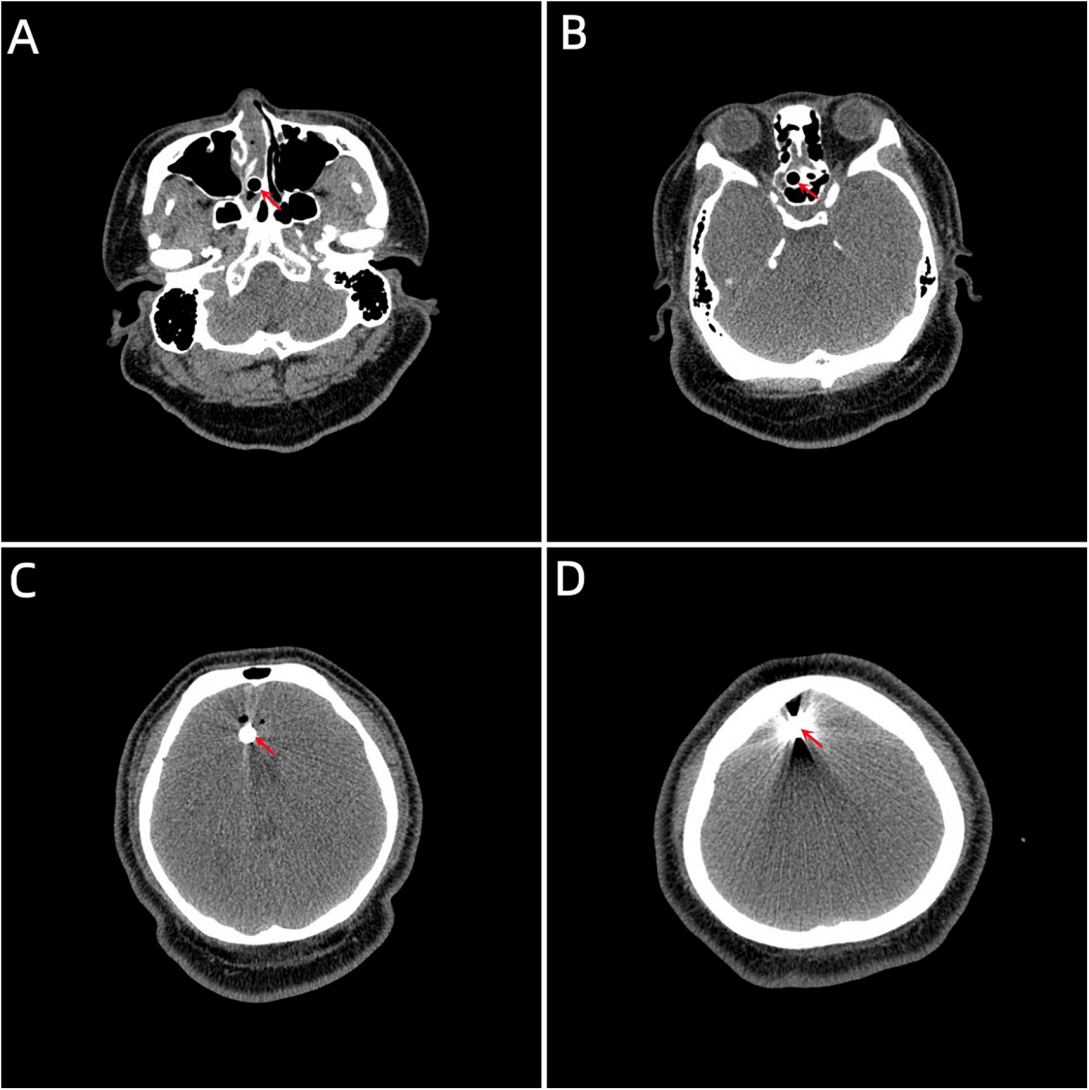
**(A)** The lower margin extending to the right nasal region through the skull base. **(B, C)** Tubular high-density shadow in the brain tissue. **(D)** The upper margin reaching the frontal bone. (the red arrow indicates the location of the foreign body location).

**Figure 3:**
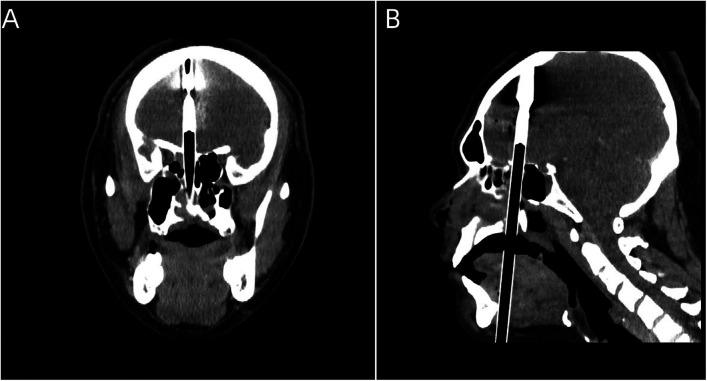
**(A)** Coronal view of the foreign body. **(B)** Sagittal view of the foreign body.

**Figure 4:**
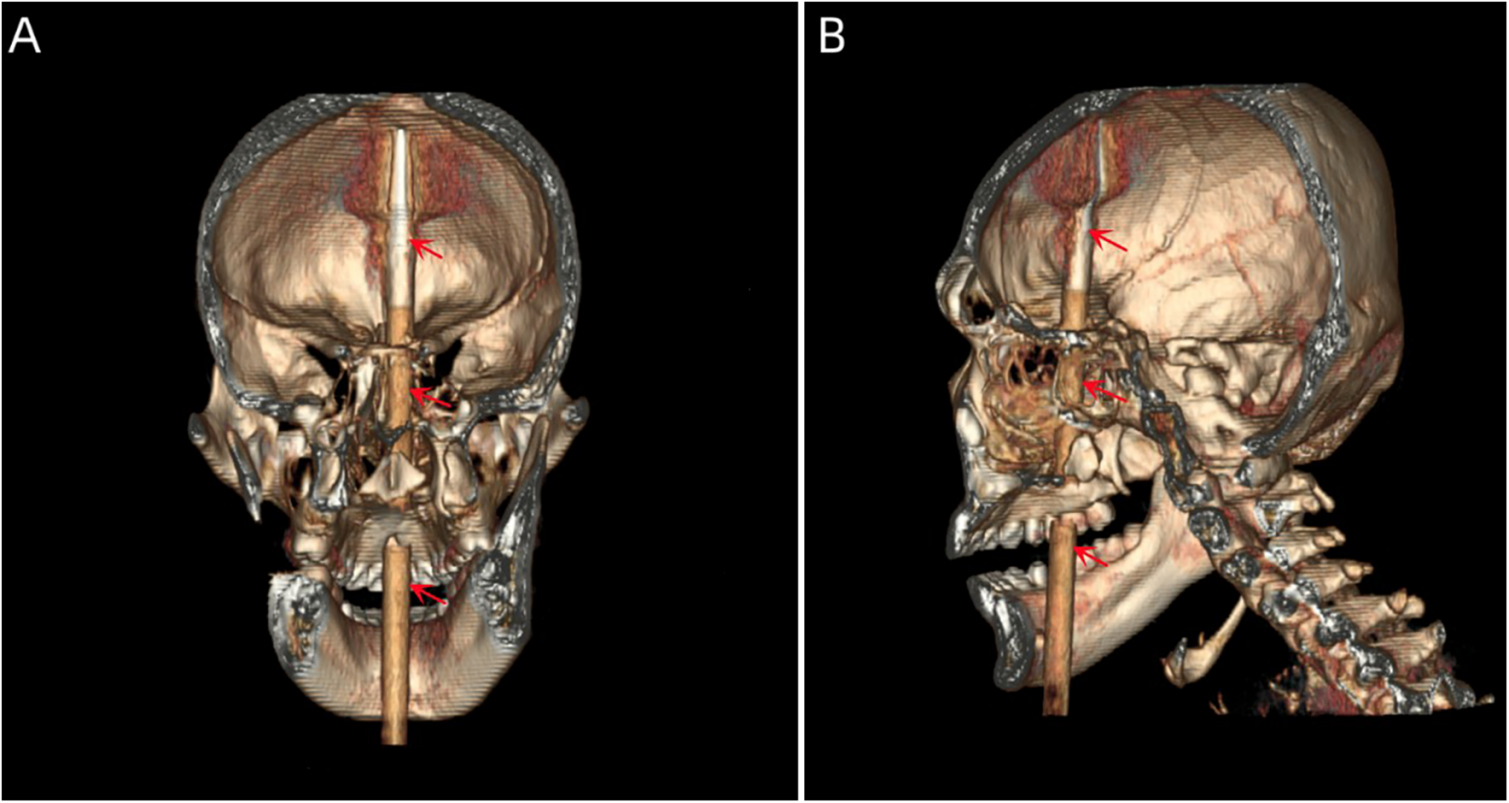
**(A, B)** 3D reconstructed images showing the location of the foreign body within the skull (the red arrow indicates the foreign body).

**Figure 5:**
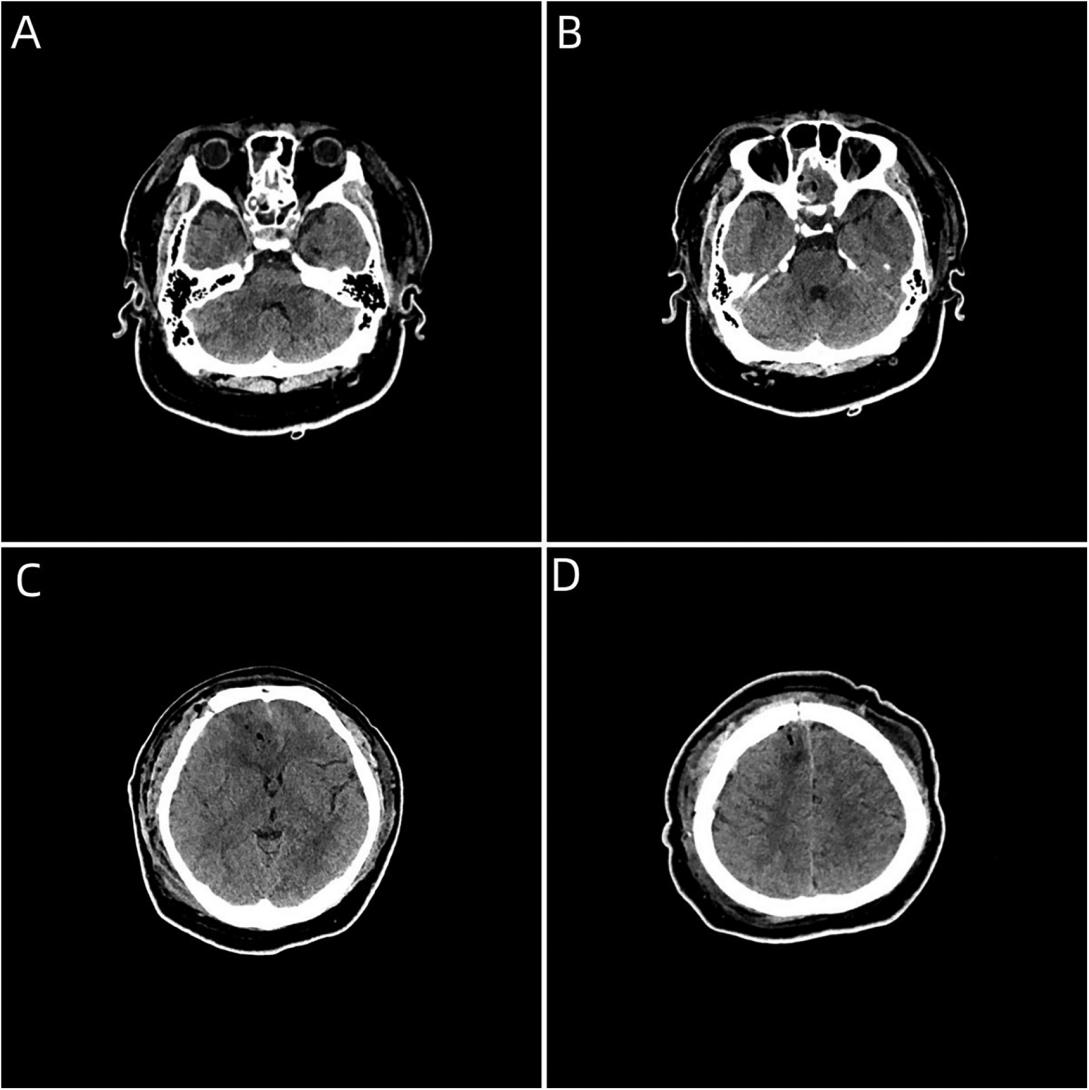
**(A–D)** Postoperative craniocerebral CT image showing complete removal of the intracranial foreign body, without foreign body residue or secondary bleeding complications.

A 20-year-old male patient was admitted to the Neurotrauma Surgery Department of Jilin University First Hospital due to attempted suicide by shooting an arrow through his mandible into his skull approximately 7 h prior. He had a 2-year history of depression, which was moderately controlled with regular oral medication. On physical examination upon admission, the patient was conscious, with a GCS score of E3VTM6. The arrow penetrated the skin and was visible in the mandibular region, and the tail of the arrow was outside the body (Figure 1A). The diameter of both pupils was 3.0 mm, and his reflexes to direct and indirect light were slow; his limb muscle strength was grade 5, his muscular tone was normal, and he had negative bilateral Babinski signs. Preoperative craniocerebral CT revealed tubular high-density shadows in the brain tissue, with the upper margin reaching the frontal bone and the lower margin extending to the right nasal region through the skull base (Figures 2, 3). Maxillofacial CT and 3D reconstruction revealed that a thin tubular foreign body had penetrated the submentum, tongue, oral cavity, right nasal cavity and ethmoid sinus, extending intracranially (Figure 4). During surgery, craniotomy was performed with a milling cutter at the transverse midline of the frontotemporal flap on the affected side. After the dura mater was exposed, local damage to the dura mater was observed, with the arrow exposed and dark red venous blood overflowing along the edge of the arrow. Bleeding was completely stopped by rinsing with hydrogen peroxide and gentamicin, after which the dura mater was cut radially. The dura mater was incised, and the subarachnoid space was opened to release cerebrospinal fluid and therefore allow slow decompression. After the collapse of the brain tissue comprising the frontal lobe, the hematoma and brain tissue damaged by the arrow were removed, the section of the arrow that extended intracranially was gradually exposed, the arrow was fixed with a bone rongeur, and the external shaft of the arrow was gently rotated until the head of the arrow was completely separated (Figure 1B) from the shaft of the arrow (Figure 1C). The head of the arrow was removed, and the shaft of the arrow was extracorpotruly retracted until it was completely removed from the skull (Figure 1D). After the damaged area of the skull base was fully exposed and the surrounding brain tissue was protected, the temporal fascia and artificial dura mater were removed to repair the damaged area of the skull base. After the skull base was completely repaired, active bleeding in the1 surgical area was 1absent, and the bone flap was also reset. The otolaryngologist subsequently continued to perform surgery to treat the nasopharyngeal injury. Postoperative craniocerebral CT reexamination revealed that the intracranial foreign body was completely removed, without residue or secondary intracranial hemorrhage (Figure 5). After surgery, the patient was administered cefminol sodium to prevent intracranial infection. He recovered well and did not exhibit signs of neurological sequelae at the 6-month follow-up visit.

## Discussion

Penetrating brain injuries are serious and have high mortality and morbidity rates (1). Arrow-induced intracranial injury is rare, and injuries caused by such weapons have historically often been accidental. However, the number of self-inflicted injuries has increased; these injuries have varying patterns and a relatively high mortality rate (1–3). PBIs caused by objects with impact velocities <100 m/s, such as metal, wood, and plastic objects, are referred to as nonfirearm penetrating brain injuries. The mechanism of craniocerebral dynamic injury is direct tearing of the injury tract (4).

Although penetrating brain injories are rarer than closed brain injuries, their prognosis is usually poor (5). These injuries mostly occur in males aged 6–55 years, with the most common cause being accidents during exercise and the second most common cause being suicide attempts (1–3, 6–8). These two causes often vary in entry location and trajectory. In accidental injuries, the arrow usually enters the skull from the frontal or occipital region. A transoral penetrating injury is a common injury associated with violent suicide (2). This type of injury is caused by gunfire (9, 10), whereas craniocerebral injury is caused by means other than gunfire (e.g., low-velocity penetrating craniocerebral injury) and is extremely rare (3). According to previous reports, 75% (12/16) of such injuries are related to suicide (Table 1). In suicide attempts, arrows usually enter through the submental or submandibular region of the mouth (1). The trajectory of the arrow is directly associated with the location of the brain injury—an injury to a major artery or the venous sinus is associated with an increased risk of vascular complications (1).

**Table 1:** Reported cases of transoral penetrating craniocerebral injury.

Reference	Age (years)	Gender	Cause	Material	Interval to operation	Antibiotics used	Infection	Outcome
Ban LH et al., (8)	43	Male	Suicide	Speargun	Not clear	Amoxicillin/Cla-vulanate	No	Left facial palsy, Cerebellar signs
Hettige S et al., (13)	38	Woman	Accident	Chopsticks	Not clear	Not clear	Not clear	Auditory and visual deterioration
Abarca-Olivas J et al., (6)	34	Male	Suicide	Speargun	2 h	Not clear	No	No neurological deficit
Sweeney JM et al., (14)	31	Male	Suicide	Knife	Not clear	Not clear	Not clear	Not clear
Khayat MA et al., (15)	31	Male	Suicide	Crossbow arrow	Not clear	Not clear	Not clear	Not clear
Aljuboori Z et al., (32)	36	Male	Suicide	Arrow	Not clear	Not clear	Not clear	No neurological deficit
	22	Male	Suicide	Arrow	Not clear	Not clear	Not clear	No neurological deficit
	67	Male	Suicide	Arrow	Not clear	Not clear	Not clear	CSF leak
Joly LM et al., (33)	42	Male	Suicide	Arrow	Not clear	Not clear	meningitis	No neurological deficit
Williams JR et al., (3)	55	Male	Suicide	Fishing harpoon gun	4.5 h	Ceftriaxone, vancomycin, metronidazole, meropenem, vancomycin	Brain abscess	Died
Bakhos D et al., (7)	35	Male	Suicide	Speargun	Not clear	Ceftriaxone, Ornidazole, amoxicillin	No	No neurological deficit
Lan ZG et al., (4)	25	Male	Accident	Metal rod	Not clear	Ceftriaxone, metronidazole	No	Not clear
Barranco R et al., (2)	59	Male	Suicide	Speargun	Not clear	Not clear	Not clear	Died
Yoneoka Y et al., (12)	65	Male	Accident	Garden pole	7 days	Piperacillin	No	No neurological deficit
Oearsakul T et al., (1)	26	Male	Suicide	Speargun	Not clear	Not clear	Not clear	No neurological deficit
Widodo D et al., (28)	28	Male	Accident	Wood	2 days	Not clear	No	No neurological deficit

Imaging examination is essential for determining the shape, size and trajectory of the penetrating foreign body, as well as to correctly diagnose the injury and select the appropriate surgical protocol (16–21). The missed detection rate of CT for nonmetallic foreign bodies is 42% (10). Several cases missed detections on CT images have been reported (16, 22, 23). The density of woody foreign bodies increases over time, possibly due to granulomas around the foreign bodies or calcium deposits inside the wood (16). Therefore, the density of foreign bodies is similar to that of brain tissue, which hinders their identification on CT. Accordingly, metallic foreign bodies are more easily diagnosed with CT, whereas MRI has great value for the identification of woody foreign bodies. Specifically, MRI helps distinguish the woody foreign body from the surrounding air and fat tissue (24). Notably, MRI may cause the movement of metal foreign bodies and may require a longer examination time. Because our patient's intracranial foreign body was made of metal (arrowhead), the patient was unable to undergo a brain MRI examination. Cerebral angiography is recommended for diagnosing transoral penetrating craniocerebral injury after ruling out cerebrovascular injury, and the association between the foreign body and the intracranial vasculature can be examined (16). Unfortunately, cerebral angiography was not performed because the equipment in our hospital was damaged on that day. CT angiography should be performed to investigate the cerebrovascular injuries either by the location or trajectory of the foreign body after an intracranial penetrating trauma. CT angiography is accurate in detecting most traumatic intracranial aneurysms, dissections, and occlusions or for revealing the location of hematomas.

Currently, clinical data are insufficient to provide an appropriate antibiotic regimen for penetrating brain injuries. Intracranial infections following penetrating brain injuries are generally associated with an increased risk of mortality (5). The rates of local wound infection, meningitis and brain abscess are elevated among patients with PBI secondary to contamination from foreign objects and skin and bone fragments introduced into the brain parenchyma along the projectile track. Before the advent of antibiotics, the rate of these infectious complications was reported to be as high as 58.8% in the military population (5). According to our review of previous cases, all but 12 patients, whose data were not explicitly recorded, were treated with antibiotics (Table 1). The infection rate was 28.6% (2/7) (9 patients did not explicitly claim to be infected) (Table 1).

Broad-spectrum antibiotics are most commonly used for treatment (16, 25). The method to select the most suitable prophylactic antibiotic regimen for patients with penetrating brain injuries varies. Cephalosporins are the most commonly used antibiotics (26). Esposito and Walker recommended treating penetrating brain injury patients with intravenous ceftriaxone, vancomycin and metronidazole for at least 6 weeks (27). Previous studies clearly indicate that all prophylactic antibiotics are broad-spectrum antibiotics (Table 1). Notably, the overuse of antibiotics can increase the risk of fungal infections. Widodo D therefore recommended the commencement of this therapeutic regimen as soon as possible after craniocerebral injury and its continuation for 5 days postoperatively (27). However, given the rarity of this condition, relevant reports are scarce; therefore, the various antibiotic treatment regimens still need further research.

The application of antiepileptic drugs has also been studied in this context. Today, the use of anticonvulsant drugs within the first 7 days of this injury is believed to be beneficial (3). However, the preventive use of anticonvulsant drugs for periods longer than 7 days is controversial. Nevertheless, the latest evidence shows that given the high incidence of epilepsy during the course of the disease, the benefits of continued use of anticonvulsant drugs are greater than their adverse effects (3).

The goal of surgical treatment for a transoral penetrating craniocerebral injury is to reduce the space-occupying effect of the foreign body and reduce the risk of infectious complications (5). Surgery is the major strategy for treating transoral penetrating craniocerebral injury (3, 26, 28, 29). Surgical indications for this injury include fracture displacement, cerebrospinal fluid leakage, foreign body residue, vascular injury and intracranial hemorrhage (3, 28, 29). However, the amount of foreign body residue or intracranial hemorrhage indicated for surgery have not been reported. Some small foreign bodies or those adjacent to important exposed structures, such as the brainstem, can be treated conservatively, and surgery should be performed within 12 h of injury (28). However, active debridement of deep fragments should be avoided because this procedure increases the risks of disability and mortality (3, 28, 29).

Early complications include vascular injury, ischemic injury, cerebral contusion hemorrhage, cerebral edema and infection, whereas late complications include hydrocephalus, cerebrospinal fluid leakage and infection (30). Early infections are associated with debris (foreign bodies, bone fragments, and hair or skin penetrating the brain) (30). Infections, including extracranial and intracranial infections such as wound infection, cranial osteomyelitis, meningitis and brain abscess, account for 5%–23% of complications. These infections are often linked to cerebrospinal fluid leakage, debris impingement and sinus wounds (30, 31). Cerebrospinal fluid leakage usually occurs as a result of dura mater tears that fail to heal. They most commonly occur at the entry wound site (or at the exit in cases of bullet injury), are present in up to 9% of patients and are highly predictive of intracranial infections (11, 30). Intranasal topical application of fluorescein is conducive to detecting cerebrospinal fluid leakage, and autologous transplantation of epifascial adipose tissue can be performed to repair cerebrospinal fluid leakage. The use of a fat-on-fascia plug shortens the surgical time, thereby reducing the risk of aerosolization during surgery (12). Specifically, minimal intranasal submucosal dissection reduces the operative time and the risk of virus “aerosolization”. In addition, the described fat-on-fascia plug method does not require the placement of a mucosal pedicle flap, which also reduces the operative time and the risk of virus aerosolization.

## Data Availability

The raw data supporting the conclusions of this article will be made available by the authors, without undue reservation.

## References

[1] Oearsakul T Kaewborisutsakul A Jantharapattana K Khumtong R Puetpaiboon A Sangthong B. Multidisciplinary management of a penetrating cerebellar injury by a fishing speargun: a case study and literature review. Surg Neurol Int. (2021) 12:391. 10.25259/SNI_506_2021 34513157 PMC8422459

[2] Barranco R Caputo F Pinto SL Drommi M Ventura F. Unusual suicide by a speargun shot: case report. Medicine (Baltimore). (2020) 99(49):e22308. 10.1097/MD.0000000000022308 33285667 PMC7717817

[3] Williams JR Aghion DM Doberstein CE Cosgrove GR Asaad WF. Penetrating brain injury after suicide attempt with speargun: case study and review of literature. Front Neurol. (2014) 5:113. 10.3389/fneur.2014.00113 25071701 PMC4083241

[4] Lan ZG Richard SA Li J Yang C. Nonprojectile penetrating iron rod from the oral cavity to the posterior cranial fossa: a case report and review of literature. Int Med Case Rep J. (2018) 11:41–5. 10.2147/IMCRJ.S157237 29563841 PMC5849932

[5] Zhu RC Yoshida MC Kopp M Lin N. Treatment of a self-inflicted intracranial nail gun injury. BMJ Case Rep. (2021) 14(1):e237122. 10.1136/bcr-2020-237122 33431447 PMC7802712

[6] Abarca-Olivas J Concepción-Aramendía LA Baño-Ruiz E Caminero-Canas MA Navarro-Moncho JA Botella-Asunción C. Perforating brain injury from a speargun. A case report. Neurocirugia (Astur). (2011) 22(3):271–5. 10.1016/S1130-1473(11)70025-2 21743951

[7] Bakhos D Villeneuve A Kim S Lebrun H Dufour X. Head spear gun injury: an atypical suicide attempt. J Craniofac Surg. (2015) 26(6):e547–8. 10.1097/SCS.0000000000002040 26335326

[8] Ban LH Leone M Visintini P Blasco V Antonini F Kaya JM Craniocerebral penetrating injury caused by a spear gun through the mouth: case report. J Neurosurg. (2008) 108(5):1021–3. 10.3171/JNS/2008/108/5/1021 18447723

[9] Nouma Y Krimi S Regaieg K. Unusual suicide by a speargun shot through the mouth: a case report. Am J Forensic Med Pathol. (2018) 39(1):73–7. 10.1097/PAF.0000000000000359 29120874

[10] Ventura F Rocca G Ventura A Celesti R. Suicide with “florbert shotgun": case report. Am J Forensic Med Pathol. (2011) 32(4):321–3. 10.1097/PAF.0b013e3181d3d2f1 20177370

[11] Kim PE Go JL Zee CS. Radiographic assessment of cranial gunshot wounds. Neuroimaging Clin N Am. (2002) 12(2):229–48. 10.1016/S1052-5149(02)00007-2 12391634

[12] Yoneoka Y Aizawa N Nonomura Y Ogi M Seki Y Akiyama K. Traumatic nonmissile penetrating transnasal anterior skull base fracture and brain injury with cerebrospinal fluid leak: intraoperative leak detection and an effective reconstruction procedure for a localized skull base defect especially after coronavirus disease 2019 outbreak. World Neurosurg. (2020) 140:166–72. 10.1016/j.wneu.2020.05.236 32497852 PMC7263210

[13] Hettige S Kok K Epaliyanage P Thomas NW. Chopstick injury penetrating the skull base: a case report. Skull Base. (2010) 20(3):219–22. 10.1055/s-0029-1246226 21318042 PMC3037098

[14] Sweeney JM Lebovitz JJ Eller JL Coppens JR Bucholz RD Abdulrauf SI. Management of nonmissile penetrating brain injuries: a description of three cases and review of the literature. Skull Base Rep. (2011) 1(1):39–46. 10.1055/s-0031-1275257 23984201 PMC3743592

[15] Khayat MA Khayat H Alhantoobi MR Aljoghaiman M Sommer DD Algird A Traumatic penetrating head injury by crossbow projectiles: a case report and literature review. Surg Neurol Int. (2024) 15:35. 10.25259/SNI_574_2023 38468667 PMC10927188

[16] Wu Y He W Yang Y Chen J. A rare case of orbitocranial penetrating injury with intracranial wooden foreign body residue. Medicina (Kaunas). (2022) 58(12):1832. 10.3390/medicina58121832 36557035 PMC9783578

[17] Wang CB Wang H Zhao JS Wu ZJ Liu HD Wang CJ Right-to-Left displacement of an airgun lead bullet after transorbital entry into the skull complicated by posttraumatic epilepsy: a case report. J Korean Neurosurg Soc. (2023) 66(5):598–604. 10.3340/jkns.2022.0234 37337741 PMC10483155

[18] Buttram SD Garcia-Filion P Miller J Youssfi M Brown SD Dalton HJ Computed tomography vs magnetic resonance imaging for identifying acute lesions in pediatric traumatic brain injury. Hosp Pediatr. (2015) 5(2):79–84. 10.1542/hpeds.2014-0094 25646200

[19] Lolli V Pezzullo M Delpierre I Sadeghi N. MDCT imaging of traumatic brain injury. Br J Radiol. (2016) 89(1061):20150849. 10.1259/bjr.20150849 26607650 PMC4985461

[20] Muehlschlegel S Ayturk D Ahlawat A Izzy S Scalea TM Stein DM Predicting survival after acute civilian penetrating brain injuries: the SPIN score. Neurology. (2016) 87(21):2244–53. 10.1212/WNL.0000000000003355 27784772 PMC5123553

[21] Vakil MT Singh AK. A review of penetrating brain trauma: epidemiology, pathophysiology, imaging assessment, complications, and treatment. Emerg Radiol. (2017) 24(3):301–9. 10.1007/s10140-016-1477-z 28091809

[22] Wilson MH Collins TR Revington PJ. Orbitocranial wooden foreign body retrieved by transcranial and superior orbitotomy. Br J Oral Maxillofac Surg. (2016) 54(9):1050–1. 10.1016/j.bjoms.2016.02.040 27475820

[23] Maruya J Yamamoto K Wakai M Kaneko U. Brain abscess following transorbital penetrating injury due to bamboo fragments–case report. Neurol Med Chir (Tokyo). (2002) 42(3):143–6. 10.2176/nmc.42.143 11936059

[24] Ramdasi R Mahore A. Bamboo in the brain—an unusual mode of injury. Indian J Surg. (2015) 77(1 Suppl):43–5. 10.1007/s12262-014-1108-4 25972640 PMC4425782

[25] Litvack ZN Hunt MA Weinstein JS West GA. Self-inflicted nail-gun injury with 12 cranial penetrations and associated cerebral trauma. Case report and review of the literature. J Neurosurg. (2006) 104(5):828–34. 10.3171/jns.2006.104.5.828 16703892

[26] Prasetyo E Oley MC Sumual V Faruk M. Transorbital-penetrating intracranial injury due to a homemade metal arrow: a case report. Ann Med Surg (Lond). (2020) 57:183–9. 10.1016/j.amsu.2020.07.049 32774851 PMC7398978

[27] Esposito DP Walker JB. Contemporary management of penetrating brain injury. Neurosurg Q. (2009) 19:249–54. 10.1097/WNQ.0b013e3181bd1d53

[28] Widodo D Perkasa F Al-'Abqary R Sjukur KJ Faruk M. Combined transcranial and transnasal endoscopic approach in transnasal-penetrating intracranial injury: a rare case report. Int J Surg Case Rep. (2022) 97:107422. 10.1016/j.ijscr.2022.107422 35872549 PMC9403176

[29] Zhang D Chen J Han K Yu M Hou L. Management of penetrating skull base injury: a single institutional experience and review of the literature. BioMed Res Int. (2017) 2017:2838167. 10.1155/2017/2838167 28828384 PMC5554568

[30] Somrani K Gader G Badri M Zammel I Rkhami M. A spectacular penetrating craniocerebral trauma from a rake: a case report. Korean J Neurotrauma. (2023) 19(1):109–14. 10.13004/kjnt.2023.19.e8 37051032 PMC10083454

[31] Rosenfeld JV Bell RS Armonda R. Current concepts in penetrating and blast injury to the central nervous system. World J Surg. (2015) 39(6):1352–62. 10.1007/s00268-014-2874-7 25446474 PMC4422853

[32] Aljuboori Z McGrath M Levitt M Moe K Chestnut R Bonow R. A case series of crossbow injury to the head highlighting the importance of an interdisciplinary management approach. Surg Neurol Int. (2022) 13:60. 10.25259/SNI_1166_2021 35242426 PMC8888290

[33] Joly LM Oswald AM Disdet M Raggueneau JL. Difficult endotracheal intubation as a result of penetrating cranio-facial injury by an arrow. Anesth Analg. (2002) 94(1):231–2, table of contents. 10.1213/00000539-200201000-00045 11772835

